# Mest but Not MiR-335 Affects Skeletal Muscle Growth and Regeneration

**DOI:** 10.1371/journal.pone.0130436

**Published:** 2015-06-22

**Authors:** Yosuke Hiramuki, Takahiko Sato, Yasuhide Furuta, M. Azim Surani, Atsuko Sehara-Fujisawa

**Affiliations:** 1 Department of Growth Regulation, Institute for Frontier Medical Sciences, Kyoto University, Kyoto, Japan; 2 Animal Resource Development Unit, RIKEN Center for Life Science Technologies, Kobe, Japan; 3 Genetic Engineering Team, RIKEN Center for Life Science Technologies, Kobe, Japan; 4 Wellcome Trust Cancer Research UK Gurdon Institute, University of Cambridge, Cambridge, United Kingdom; 5 Department of Physiology, Development and Neuroscience, University of Cambridge, Cambridge, United Kingdom; 6 Wellcome Trust-Medical Research Council Stem Cell Institute, University of Cambridge, Cambridge, United Kingdom; Institut de Myologie, FRANCE

## Abstract

When skeletal muscle fibers are injured, they regenerate and grow until their sizes are adjusted to surrounding muscle fibers and other relevant organs. In this study, we examined whether *Mest*, one of paternally expressed imprinted genes that regulates body size during development, and miR-335 located in the second intron of the *Mest* gene play roles in muscle regeneration. We generated miR-335-deficient mice, and found that miR-335 is a paternally expressed imprinted microRNA. Although both *Mest* and miR-335 are highly expressed during muscle development and regeneration, only *Mest^+/-^* (maternal/paternal) mice show retardation of body growth. In addition to reduced body weight in *Mest^+/^-; DMD-null* mice, decreased muscle growth was observed in *Mest^+/-^* mice during cardiotoxin-induced regeneration, suggesting roles of Mest in muscle regeneration. Moreover, expressions of *H19* and *Igf2r*, maternally expressed imprinted genes were affected in tibialis anterior muscle of *Mest^+/-^; DMD-null* mice compared to *DMD-null* mice. Thus, Mest likely mediates muscle regeneration through regulation of imprinted gene networks in skeletal muscle.

## Introduction

Skeletal muscle increases its mass during fetal, neonatal, and juvenile development. It has been known that IGF1-AKT-mTOR and Myostatin-Smad pathways are involved in positive and negative regulation of skeletal muscle mass, respectively [1]. These signaling pathways likely undertake muscle mass maintenance and plasticity in adult in addition to regulation of muscle growth during development. On the other hand, lengths of skeletal muscle fibers should be coordinately regulated with other relevant organs such as bones and tendons during development. Although underlying genetic and epigenetic mechanisms and gene networks for such a coordinated growth of skeletal muscle with body size still need to be elucidated, genetic evidence suggests that many genomic imprinted genes are involved in that process [2–8]. Under this scenario, some of these imprinted genes should also regulate muscle regeneration in which muscle stem cells expand and fuse with each other or with damaged myofibers to form adequate-sized regenerating muscle that fits surrounding tissues.

In this study, we examined whether the *Mest* (Mesoderm specific transcript) gene, a paternally expressed imprinted gene that regulates body size and miR-335, a microRNA (miRNA) located in intron 2 of *Mest*, are involved in muscle regeneration. miRNAs are a class of small noncoding RNAs of ~23 nucleotides that inhibit gene expression by binding 3’UTR of target mRNAs, and have emerged as a class of key post-transcriptional regulators of gene expression [9,10]. Recent studies have revealed the involvement of miRNAs in elaborate regulation of cell proliferation and differentiation [11]. We sought to determine whether miR-335 was co-regulated with Mest during postnatal development and skeletal muscle regeneration. When Mest-deficient mice were generated previously, miR-335 had not been identified in the *Mest* locus [2]. Although miR-335 genomic locus was left intact in the *Mest* ^*+/-*^ (maternal/paternal) mice, our finding that expression of miR-335 was significantly decreased in those mice lead us to examine which is responsible for the regulation of body size, Mest, miR-335, or both. miR-335 mutant mice revealed that Mest, but not miR-335, is responsible for the control of body and muscle sizes and for the regulation of muscle growth during regeneration. Although miR-335 was co-regulated with Mest as a paternally expressed imprinted miRNA, neither body growth nor muscle regeneration was affected by deleting miR-335.

Two types of experimental muscle regenerating models were used in this study. One is to analyze the acute phase of regeneration induced by cardiotoxin (CTX) administration into skeletal muscle, and the other is to investigate the chronic phase of regeneration in *Dystrophin* gene-deleted (*DMD-null*) mice [12], useful model for Duchenne Muscular Dystrophy (DMD). DMD is one of the most common and severe forms of muscular dystrophy [13–15]. DMD muscle fibers are highly susceptible to mechanical damage and therefore exhibit chronic or repetitive regeneration. *Mest* ^*+/-*^ *; DMD-null* mice exhibited a significant reduction in body growth after 1 week compared to *DMD-null* mice, and showed the dysregulation of *H19* and *Igf2r* genes, maternally expressed imprinted genes. Moreover *Mest* ^*+/-*^ mice resulted in decrease in muscle fiber thickness when skeletal muscle regeneration was induced with CTX. Roles of Mest in muscle regeneration are discussed based on these results.

## Results

### *Mest* and miR-335 are coordinately expressed during skeletal muscle development and regeneration

**Fig 1:**
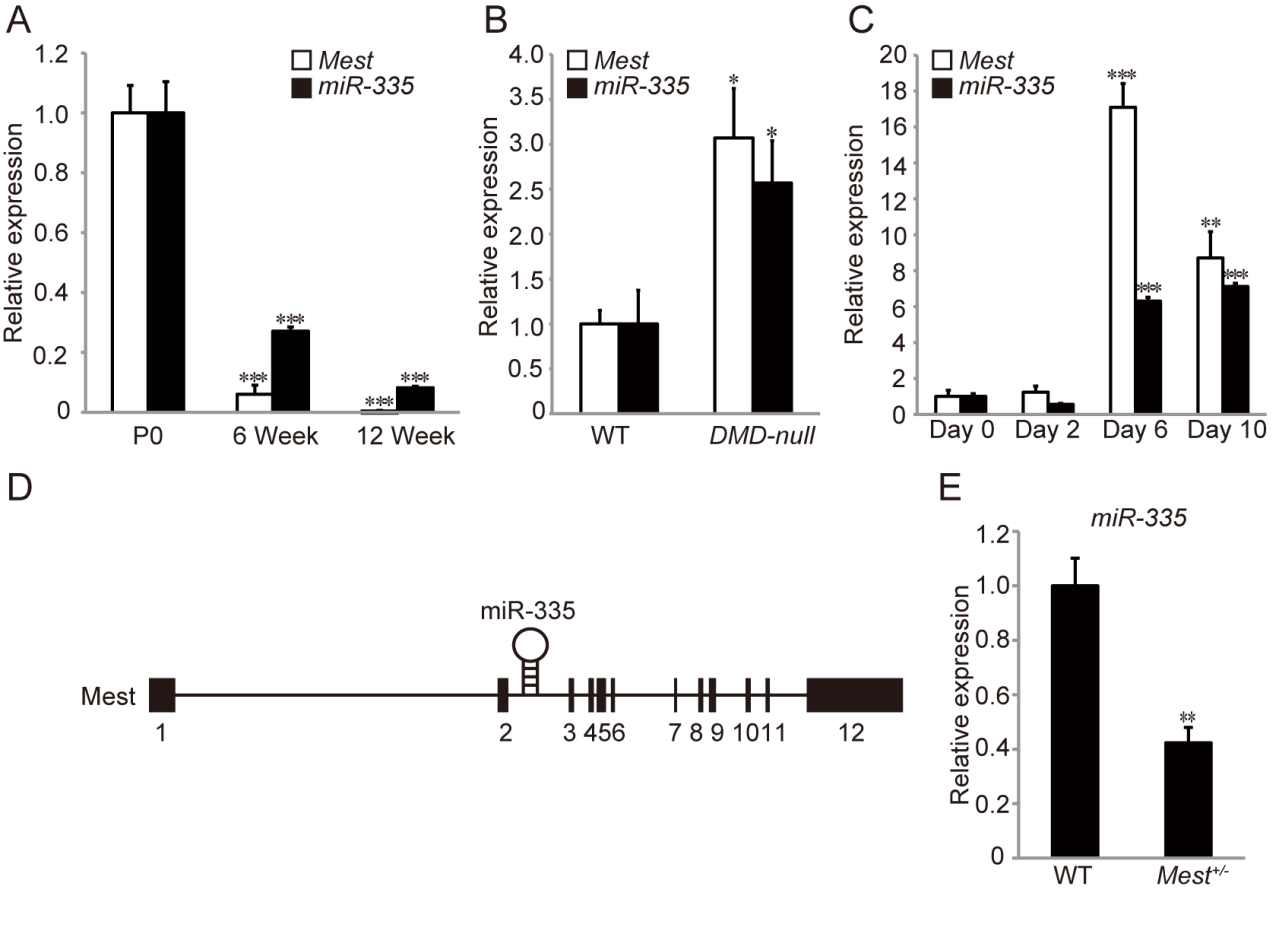
*Mest* and miR-335 are coordinately expressed in skeletal muscle during postnatal development and regeneration. (A) qRT-PCRs for *Mest* mRNA and miR-335 were performed with TA muscles of P0, 6 weeks, and 12 weeks old WT mice (n = 3 per time point). (B) qRT-PCRs for *Mest* mRNA and miR-335 were performed with TA muscles of 3 months old WT (n = 4) and *DMD–null* mice (n = 7). (C) qRT-PCRs for *Mest* mRNA and miR-335 were performed with TA muscles from day 0 to day 10 after CTX injection (n = 3 per time point). (D) A schematic diagram of the Mest and miR-335 genomic region on chromosome 6 in mouse. (E) qRT-PCR for miR-335 was performed in TA muscles of WT and *Mest* ^*+/-*^ mice (n = 3 per genotype). Expression of *Mest* and that of miR-335 are normalized to *Gapdh* and snoRNA-202, respectively. Error bars indicate the s.e.m. *P < 0.05, **P < 0.01, ***P < 0.001 compared with P0 (A), WT (B and E), and day 0 (C).

Both *Mest* and miR-335 were highly expressed in tibialis anterior (TA) muscles at postnatal day 0 (P0), and decreased gradually as mice grew up (Fig 1A). A previous report showed that miR-335 was up-regulated in adductor muscle of *mdx* mice and skeletal muscle biopsy of DMD patients in common [16]. We found the up-regulation of *Mest* as well as miR-335 in TA muscles of 3 months old *DMD–null* compared with that of wild type (WT) mice (Fig 1B). Since *DMD-null* mice undergo repetitive myofiber degeneration and regeneration, we next examined whether *Mest* and miR-335 were up-regulated either in degenerating or in regenerating phases upon skeletal muscle injury induced by the administration of CTX into TA muscles. Degenerating and regenerating muscles were isolated 2 days and 6–10 days after the CTX injection, respectively. Both *Mest* and miR-335 were remarkably activated in the regenerating phase but not in the degenerating phase (Fig 1C). Thus, *Mest* and miR-335 are expressed coordinately during skeletal muscle growth and regeneration, and in the pathological condition of DMD. The Mest mutant mice had been established by deleting exons 3–8 and part of exon 9 previously [2], which left the miR-335 genomic locus locating within the intron 2 of the *Mest* gene intact (Fig 1D). Interestingly, however, quantitative RT-PCR (qRT-PCR) analyses revealed that expression of miR-335 was significantly lower in *Mest* ^*+/-*^ than in WT mice (Fig 1E).

### miR-335 is a paternally expressed imprinted gene

**Fig 2:**
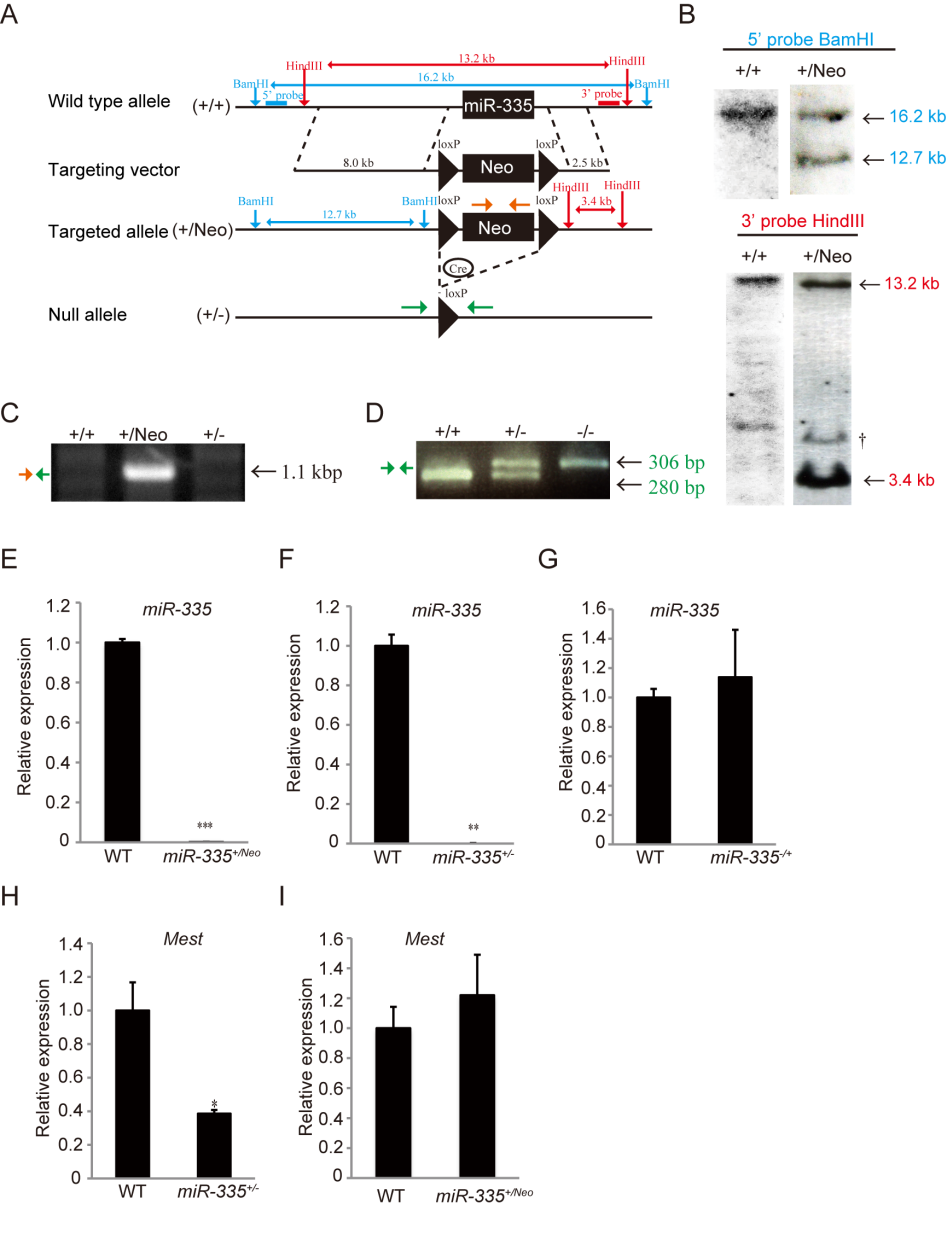
Generation of miR-335 deficient mice. (A) Design of constructs used for generation of miR-335 deficient mice. The miR-335 genomic locus was replaced by a floxed neomycin-resistance cassette (loxP-Neo-loxP) to obtain *miR-335* ^*+/Neo*^ mice. *miR-335* ^*+/-*^ mice (+/-) were generated by crossing male *miR-335* ^*+/Neo*^ mice with female CAG-*cre* transgenic mice. (B) Southern blot analysis of WT and G418 resistant ES clones with 5’ and 3’ probes. †: Non-specific band. (C and D) PCR analysis for targeted allele with genomic DNA in tails of WT (+/+), *miR-335* ^*+/Neo*^ (+/Neo), and *miR-335* ^*+/-*^ mice (+/-). In (C), an insertion and a deletion of a floxed neomycin-resistance cassette in genomic DNA of *miR-335* ^*+/Neo*^ (+/Neo) and *miR-335* ^*+/-*^ mice (+/-), respectively, were detected with PCRs amplified with primers shown in Fig 2A (Orange and Green arrows). In (D), a PCR analysis to distinguish alleles for WT (+/+), *miR-335* ^*+/-*^ (+/-), and *miR-335* ^*-/-*^ mice (-/-) was shown. The WT allele-specific (280 bp) and the mutant allele-specific (306 bp) bands were amplified with the primers shown in Fig 2A (Green arrow). (E, F and G) qRT-PCR for miR-335 was performed in TA muscles isolated from WT, *miR-335* ^*+/Neo*^, and *miR-335* ^*+/-*^ or *miR-335* ^*-/+*^ mice (n = 3 per genotype). (H and I) qRT-PCR for *Mest* mRNA was performed in TA muscles of WT, *miR-335* ^*+/-*^, and *miR-335* ^*+/Neo*^ mice (n = 3 per genotype). Expression of *Mest* mRNA and that of miR-335 are normalized to *Gapdh* and snoRNA-202, respectively. Error bars indicate the s.e.m. *P < 0.05, **P < 0.01, ***P < 0.001.

To distinguish roles of Mest from those of miR-335, we generated miR-335 deficient mice by homologous recombination in embryonic stem (ES) cells. The miR-335 sequence was replaced with a neomycin resistance cassette flanked by loxP sites (Fig 2A). Targeted ES clones were confirmed by Southern blotting using 5’ and 3’ probes against mouse genomic DNA digested with BamHI and HindIII, respectively (Fig 2B). The absence of random integration was verified with a neomycin probe (data not shown). Removal of the neomycin resistance cassette by mating male *miR-335* ^*+/Neo*^ mice to female CAG-*cre* transgenic mice resulted in the deletion of short flanking sequences together with the neomycin cassette (*miR-335* ^*+/-*^ mice) (Fig 2C and 2D). *Mest* is an imprinted gene that is expressed essentially from the paternal allele during development [2]. We investigated whether miR-335 is also paternally expressed. qRT-PCR analysis revealed that *miR-335* ^+/Neo^ (maternal/paternal) mice obtained by crossing male *miR-335* ^+/Neo^ mice with female WT mice scarcely expressed miR-335 in skeletal muscle (Fig 2E). Similarly, *miR-335* ^+/-^ (maternal/paternal) mice obtained by crossing male *miR-335* ^+/-^ mice with female WT mice scarcely expressed miR-335 in skeletal muscle (Fig 2F). On the contrary, *miR-335* ^-/+^ (maternal/paternal) mice obtained by crossing male WT mice with female *miR-335* ^+/-^ mice expressed miR-335 in skeletal muscle comparable to WT mice (Fig 2G). Thus, these results indicate that miR-335 is a paternally expressed imprinted miRNA. In addition, while the expression of *Mest* was decreased in *miR-335* ^*+/-*^ mice, it was not altered in *miR-335* ^*+/Neo*^ mice (Fig 2H and 2I), suggesting that the region surrounding the neomycin resistance cassette flanked by loxP sites, but not the miR-335 itself, is involved in transcriptional regulation of *Mest*. To evaluate roles of miR-335, further analyses were mainly performed with *miR-335* ^*+/Neo*^ mice.

### Mest but not miR-335 affects body growth

**Fig 3:**
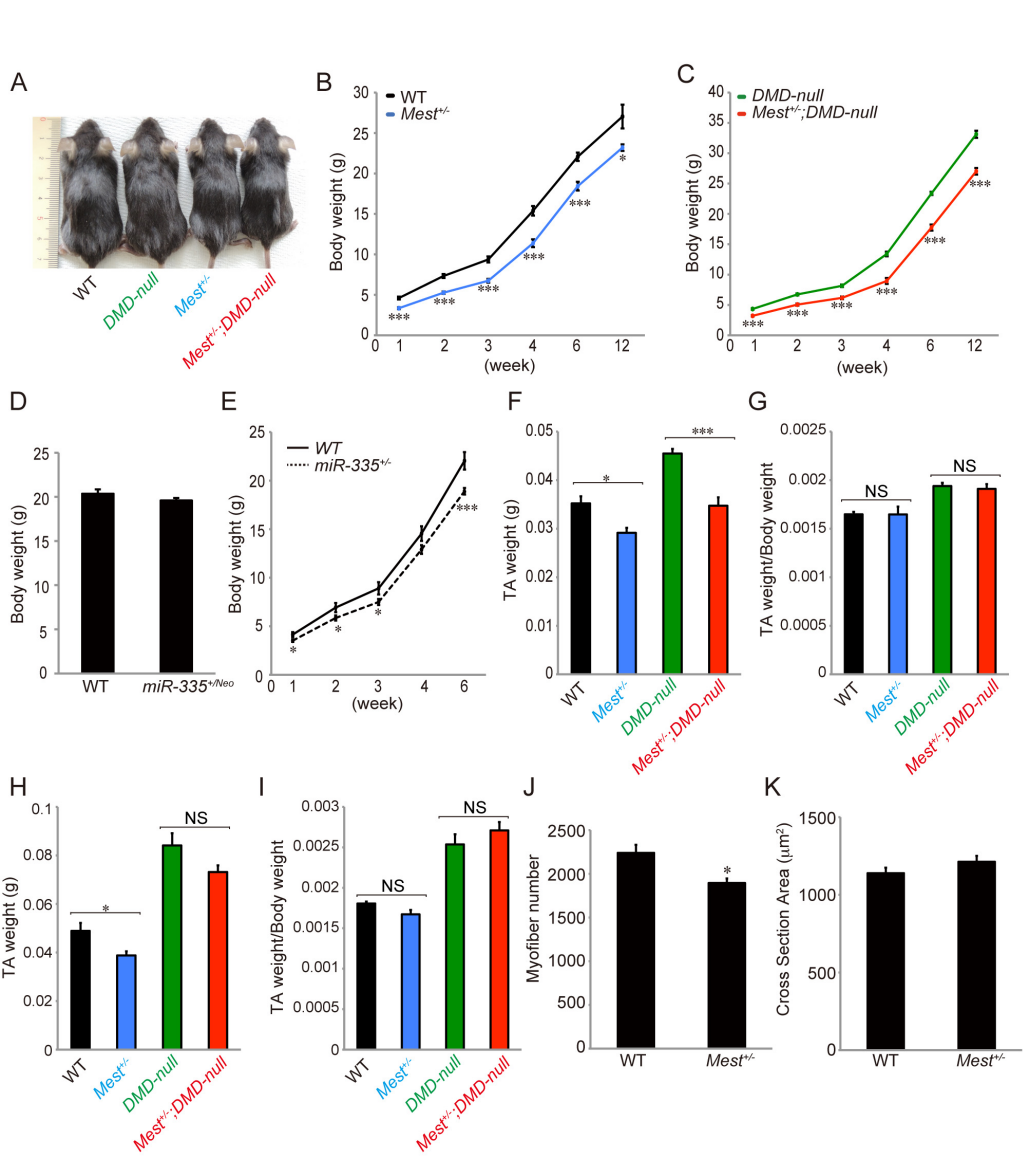
Mest is required for body and skeletal muscle growth. (A) Representative images of 4 weeks old mice in individual genotypes. (B and C) Body weights of male littermate WT (n = 3–22), *Mest* ^*+/-*^ (n = 6–15), *DMD-null* (n = 4–25), and *Mest* ^*+/-*^ *; DMD-null* mice (n = 4–22) from 1 to 12 (11–13) weeks old. (D) Body weights of WT (n = 15) and *miR-335* ^*+/Neo*^ (n = 12) mice at 6 weeks. (E) Body weights of WT (n = 8–15) and *miR-335* ^*+/-*^ mice (n = 15–18) from 1 to 6 weeks old. (F) TA muscle weights of male littermate WT (n = 13), *Mest* ^*+/-*^ (n = 6), *DMD-null* (n = 18), and *Mest* ^*+/-*^ *; DMD-null* mice (n = 11) at 6 weeks old. (G) TA/Body weights of male littermate WT, *Mest* ^*+/-*^, *DMD-null*, and *Mest* ^*+/-*^ *; DMD-null* mice at 6 weeks old. (H) TA muscle weights of male littermate WT (n = 3), *Mest* ^*+/-*^ (n = 6), *DMD-null* (n = 4), and *Mest* ^*+/-*^ *; DMD-null* mice (n = 4) at 11–13 weeks old. (I) TA/Body weights of male littermate WT, *Mest* ^*+/-*^, *DMD-null*, and *Mest* ^*+/-*^ *; DMD-null* mice at 11–13 weeks old. (J and K) The numbers and average cross section areas of TA muscle fibers of male littermate WT (n = 7) and *Mest* ^*+/-*^ mice (n = 4) at 6 weeks. Error bars indicate the s.e.m. *P < 0.05, ***P < 0.001. NS = Not significant.

*Mest* ^*+/-*^ mice show growth retardation ([2], Fig 3A and 3B). *miR-335* ^*+/Neo*^ mice, in contrast, did not show a reduction in body weight compared to WT mice significantly (Fig 3D). *miR-335* ^*+/-*^ mice exhibited mild growth defects (Fig 3E), which is likely due to decreased expression of Mest in *miR-335* ^*+/-*^ mice (Fig 2H). Based on these results, we concluded that Mest, but not miR-335, is involved in regulation of body size during development. When TA muscles of WT and *Mest* ^*+/-*^ mice were compared at 6 and 11–13 weeks of age, those of the latter were significantly smaller than those of the former (Fig 3F and 3H). Moreover, numbers of muscle fibers are significantly less in *Mest* ^*+/-*^ mice (Fig 3J). In contrast, the ratio of TA weight/Body weight and the average cross section areas of TA muscles of both WT and *Mest* ^*+/-*^ mice were similar to each other (Fig 3G, 3I and 3K).

### Mest affects skeletal muscle growth during regeneration

**Fig 4:**
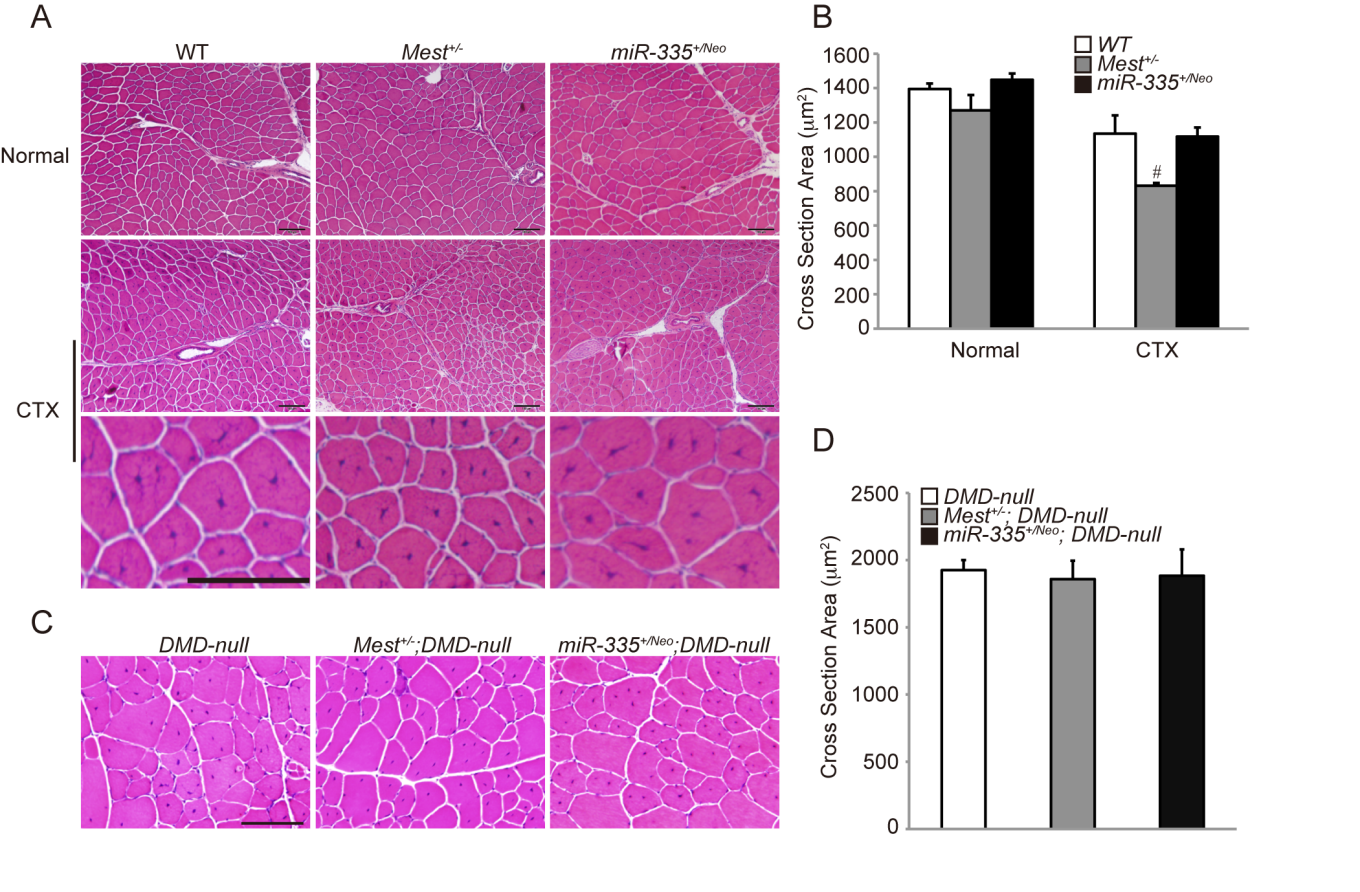
Mest is required for skeletal muscle growth during regeneration. (A) H&E staining of TA muscles under normal condition (top panel) and 14 days after CTX-induced injury (middle and bottom panels). Bottom panel shows the extended images of a part of middle panel. (B) Average cross section areas of TA muscles in WT (n = 3), *Mest* ^*+/-*^ (n = 4) and *miR-335* ^*+/Neo*^ mice (n = 6) under normal condition and WT (n = 7), *Mest* ^*+/-*^ (n = 4) and *miR-335* ^*+/Neo*^ mice (n = 6) 14 days after CTX injury. (C) H&E staining of TA muscles of *DMD-null*, *Mest* ^*+/-*^ *; DMD-null* and *miR-335* ^*+/Neo*^ *; DMD-null* mice at 11–13 weeks old. (D) Average cross section areas of TA muscles in *DMD-null* (n = 8), *Mest* ^*+/-*^ *; DMD-null* (n = 4) and *miR-335* ^*+/Neo*^ *; DMD-null* mice (n = 4) at 11–13 weeks old. Error bars indicate the s.e.m. ^*#*^ *P* = 0.0549 compared with WT mice. Scale bar: 100 μm.

Although the expression of *Mest* is up-regulated in regenerating skeletal muscle induced by injection of CTX and in skeletal muscle of adult *DMD–null* mice (Fig 1B and 1C), roles of Mest in muscle regeneration and pathology of DMD remain unknown. In order to clarify roles of Mest in muscle regeneration, we examined whether muscle regeneration induced by CTX was affected in *Mest* ^*+/-*^ mice. Ratios of myofibers with central nuclei in *Mest* ^*+/-*^ mice were comparable to those in WT mice, indicating that muscle regeneration occurs in the absence of Mest (Fig 4A). However, regenerating muscle fibers were significantly thinner in *Mest* ^*+/-*^ than those in WT mice when analyzed 14 days after CTX-injection while average cross section areas of intact TA muscle fibers of both WT and *Mest* ^*+/-*^ mice were similar to each other (Fig 4A and 4B). These results indicate that Mest is required for efficient muscle growth during muscle regeneration. Next, we investigated whether Mest affects pathology of *DMD-null* mice. There is no significant difference in Cross Section Areas (CSA) between *DMD-null* and *Mest* ^*+/-*^ *; DMD-null* mice (Fig 4C and 4D). We analyzed body weight of *DMD-null* and *Mest* ^*+/-*^ *; DMD-null* mice. Fig 3C shows that body weights of *Mest* ^*+/-*^ *; DMD-null* mice were consistently smaller than those of *DMD-null* mice from 1 week to 11–13 weeks. If Mest promotes muscle regeneration in addition to body growth during postnatal development, the difference in body weights between *DMD-null* and *Mest* ^*+/-*^ *; DMD-null* mice should be accelerated after muscle degeneration and regeneration start in them. In contrast, such an accelerated difference in body weights would not be observed between WT and *Mest* ^*+/-*^ mice, which no regeneration occurs. As shown in S1 Fig, the ratio of *Mest* ^*+/-*^ *; DMD-null/ DMD-null* indeed became slightly lower than that of *Mest* ^*+/-*^/WT after 4 weeks of age when muscle degeneration and regeneration start in those mice. The result implies contribution of Mest in body growth during muscle regeneration as well as contribution in body growth during postnatal development. Furthermore, we analyzed the relation between body and TA weight in 6 and 11–13 weeks *DMD-null* and *Mest* ^*+/-*^ *; DMD-null* mice. TA weights of *Mest* ^*+/-*^ *; DMD-null* mice were significantly smaller at 6 weeks and slightly smaller at 11–13 weeks compared to those of *DMD-null* mice although statistical significance could not be obtained with the latter probably due to small numbers of mice we analyzed (Fig 3F and 3H). TA/body weight was more or less constant among these mice (Fig 3G and 3I). In contrast, there was no significant difference in muscle growth during regeneration induced by CTX between WT and *miR-335* ^*+/Neo*^ mice and in growth of Dystrophin-deficient muscle between *DMD-null* and *miR-335* ^*+/Neo*^ *; DMD-null* mice (Fig 4A–4D). Therefore, these result suggest that Mest but not miR-335 is involved in skeletal muscle growth during regeneration.

### *H19* and *Igf2r* are affected in *Mest* ^*+/-*^ *; DMD-null* mice

**Fig 5:**
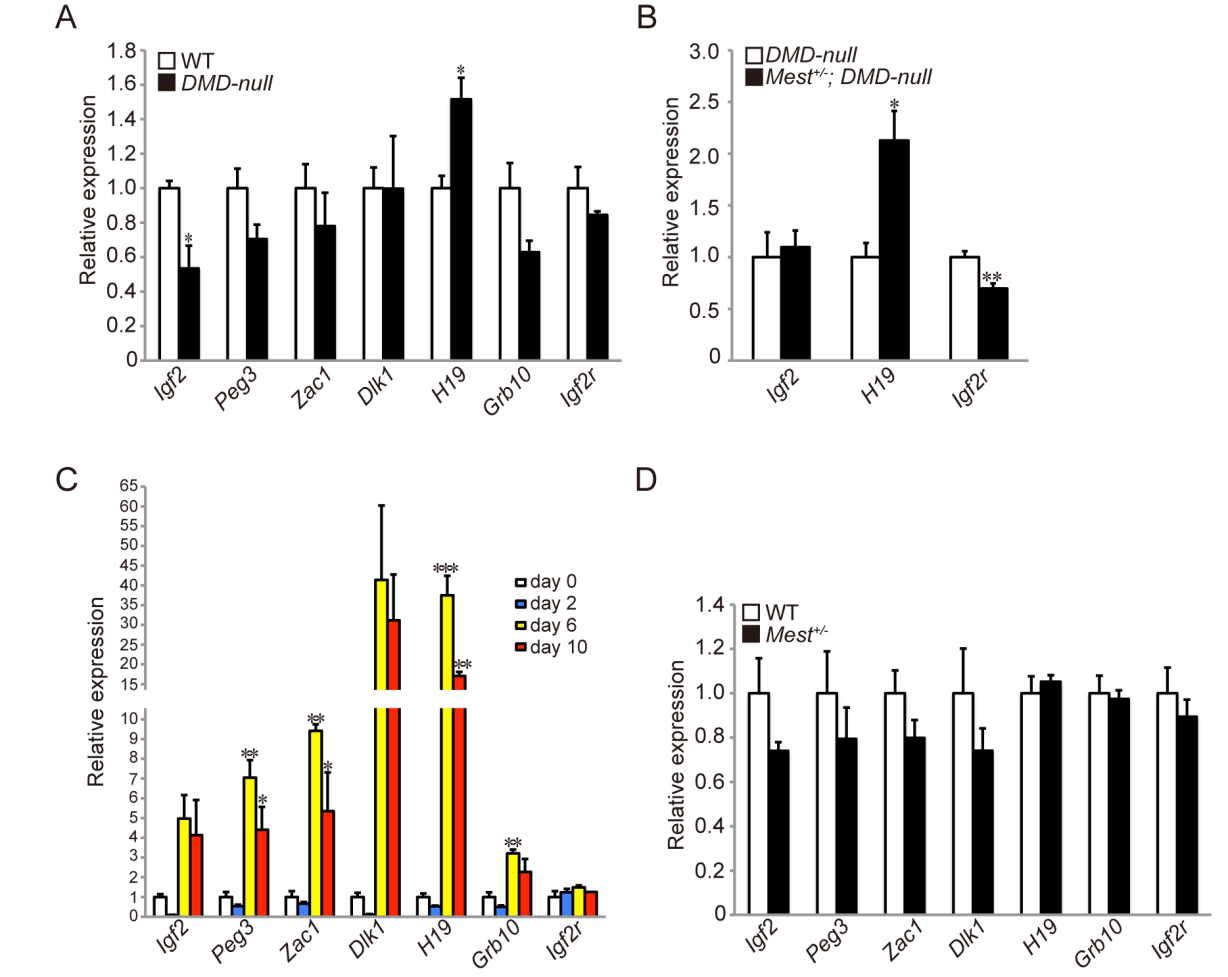
The expression level of imprinted genes is altered in *DMD-null* mice and CTX injury. (A) qRT-PCRs for paternally expressed (*Igf2*, *Peg3*, *Zac1*, *Dlk1*) and maternally expressed (*H19*, *Grb10*, *Igf2r*) imprinted genes were performed in TA muscles of 6 weeks old WT and *DMD-null* mice (n = 3 per genotype). (B) qRT-PCRs for *Igf2*, *H19*, and *Igf2r* mRNA were performed in TA muscles of 6 weeks old *DMD-null* (n = 4) and *Mest* ^*+/-*^ *; DMD-null* (n = 6). (C) qRT-PCRs for imprinted genes were performed with TA muscles obtained from WT mice from day 0 to day 10 after CTX injection (n = 3 per time point). (D) qRT-PCRs for imprinted genes were performed in TA muscles of 12–15 weeks old WT (n = 5) and *Mest* ^*+/-*^ mice (n = 3) 6 days after CTX. Error bars indicate the s.e.m. *P < 0.05, **P < 0.01, ***P < 0.001.

Imprinted gene networks have been reported to control somatic growth [5, 17, 18]. Such imprinted gene networks might also regulate growth of muscle fibers during skeletal muscle regeneration. Indeed *Dlk1*, a paternally expressed imprinted gene, is involved in skeletal muscle regeneration [19]. To investigate whether Mest has the relation with some paternally and maternally expressed imprinted genes in regenerating TA muscles, we focused on paternally (*Igf2*, *Peg3*, *Zac1*, and *Dlk1*) and maternally (*H19*, *Grb10*, and *Igf2r*) expressed imprinted genes. qRT-PCR revealed that the expression of *Igf2* and *H19* was altered in TA muscles at 6 weeks old *DMD–null* mice compared with WT mice (Fig 5A). Expression of *H19* was further augmented while expression of *Igf2* was not affected in *Mest* ^*+/-*^ *; DMD-null* mice (Fig 5B). Instead, *Igf2r* expression was attenuated in *Mest* ^*+/-*^ *; DMD-null* mice (Fig 5B). Thus, *H19* and *Igf2r*, maternally expressed imprinted genes, were affected in pathologically regenerating muscle of *Mest* ^*+/-*^ *; DMD-null* mice. Next, we examined whether some imprinted genes were up-regulated either in degenerating or regenerating phase upon skeletal muscle injury by CTX. These genes except for *Igf2r* were remarkably activated in the regenerating phase (Fig 5C). However, there was no significant difference based on the expression of these imprinted genes between WT and *Mest* ^*+/-*^ mice at 6 days after CTX (Fig 5D).

### Muscle satellite cells are not affected in *Mest* ^*+/-*^ mice

**Fig 6:**
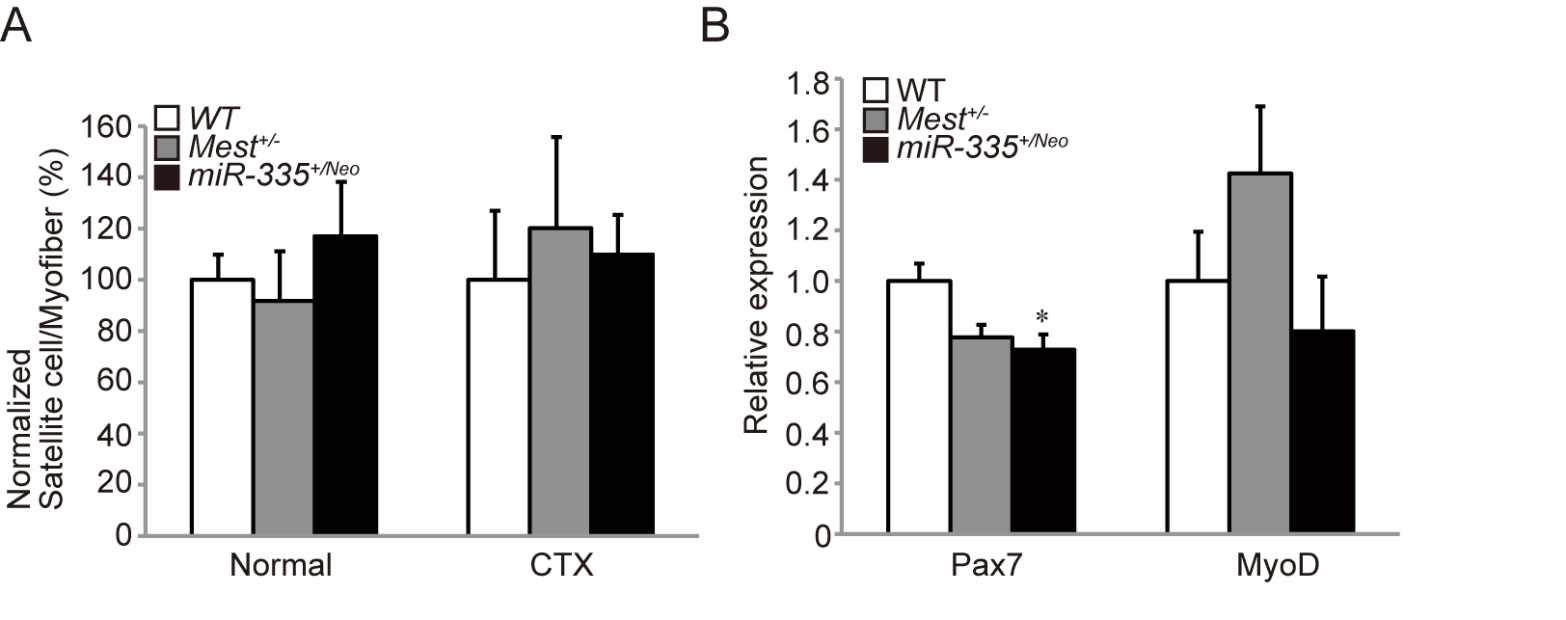
Muscle satellite cells are not affected in *Mest* ^*+/-*^ mice. (A) Numbers of Pax7 positive-satellite cells per muscle fiber in sections of TA muscles of WT, *Mest* ^*+/-*^, and *miR-335* ^*+/Neo*^ mice (n = 3 per genotype) at 10 weeks old in normal condition and 14 day after CTX. (B) qRT-PCRs for *Pax7* and *MyoD* mRNA were performed in normal TA muscles of 11–13 weeks old WT, *Mest* ^*+/-*^, and *miR-335* ^*+/Neo*^ mice (n = 3 per genotype). Error bars indicate the s.e.m. *P < 0.05 compared with WT mice.

The decrease of TA weight and the thin CSA in *Mest* ^*+/-*^ mice could be due to some defects in muscle satellite cells, skeletal muscle-specific stem cells. In order to investigate functions of Mest in muscle satellite cells, we compared numbers of muscle satellite cells in TA muscles of WT and *Mest* ^*+/-*^ mice in normal and CTX injured condition. As a result, there was no significant difference in numbers of Pax7-positive muscle satellite cells per myofiber between WT and *Mest* ^*+/-*^ mice (Fig 6A). Next, in order to evaluate maintenance and differentiation of muscle satellite cells, we examined whether expressions of Pax7 for maintenance and MyoD for differentiation are affected in *Mest* ^*+/-*^ mice. As a result, there was no significant difference for expression of *Pax7* and *MyoD* both in normal (Fig 6B) and CTX injured condition (S2 Fig) in *Mest* ^*+/-*^ mice compared to WT mice. In addition, although *miR-335* ^*+/Neo*^ mice show no significant difference in number of Pax7-positive muscle satellite cells per myofiber, the expression of Pax7 was decreased (Fig 6A and 6B).

## Discussion

### Mest is a positive regulator of muscle growth during development and regeneration

In this study, we showed roles of the Mest gene in control of body size and skeletal muscle growth during postnatal development and regeneration. Because the *Mest* includes miR-335 in its second intron, we examined a possibility whether miR-335 is also involved in body and skeletal muscle growth and in muscle regeneration. Although *Mest* ^*+/-*^ mice have miR-335 genomic locus, the expression of miR-335 was down-regulated in *Mest* ^*+/-*^ mice compared to WT mice (Fig 1E). Thus, it was necessary to analyze miR-335 deficient mice to conclude that the *Mest* gene, not miR-335, is responsible for control of body growth. *miR-335* ^*+/Neo*^ mice showed normal body weight and expression of *Mest* while *miR-335* ^*+/-*^ mice showed reduced body weight and expression of *Mest* (Figs 2H, 2I, 3D and 3E). These results suggest that decreased expression of *Mest* in *miR-335* ^*+/-*^ mice, but not the deletion of miR-335 itself, causes reduced body weight.

Although previous studies pointed out high-level expression of *Mest* together with other imprinted genes in skeletal muscle [20], its roles in muscle growth and regeneration have been elusive. It is noteworthy that the average cross section areas in *Mest* ^*+/-*^ mice was significantly smaller in CTX-induced regeneration model, but not in *Mest* ^*+/-*^ *; DMD-null* mice at 11–13 weeks. Mest was much more prominently enhanced in muscle after CTX injection than DMD muscle (Fig 1B and 1C). Therefore, different molecular mechanisms could regulate skeletal muscle regeneration induced by CTX and by Dystrophin-deficiency. Alternatively, synchronous progression of CTX-induced regeneration could be affected in *Mest* ^*+/-*^ mice more clearly than regeneration caused by Dystrophin-deficiency that occurs randomly within muscle tissues. Although the decrease of TA weight and the thin CSA in injured *Mest* ^*+/-*^ mice are observed (Figs 3F, 3H and 4B), there was no significant difference in numbers of Pax7-positive muscle satellite cells per myofiber between WT and *Mest* ^*+/-*^ mice (Fig 6A), suggesting that quiescent satellite cells are generated and maintained similarly in WT and *Mest* ^*+/-*^ mice. Moreover, *Pax7* and *MyoD* genes were similarly expressed in normal (Fig 6B) and CTX-induced injured conditions (S2 Fig) in *Mest* ^*+/-*^ mice compared to those in WT mice, suggesting that expression of myogenic transcription factors in muscle satellite cells was not affected by the lack of Mest during CTX-induced myogenic activation. Thus, the reduction of muscle growth by the lack of Mest in CTX-induced regeneration model is likely due to decreased proliferation of muscle satellite cells after their activation and/or decreased thickening of muscle fibers after myotube formation. Taken similarity in CSAs of intact TA muscle fibers of both WT and *Mest* ^*+/-*^ mice into account, involvement of Mest in proliferation of muscle satellite cells rather than that in hypertrophic growth of muscle fibers is favorable for the interpretation of these data. In contrast, expression of Pax7 was affected in *miR-335* ^*+/Neo*^ mice. However, more precise analyses need to be performed in order to know whether proliferation and differentiation of muscle satellite cells during muscle regeneration are affected in *Mest* ^*+/-*^ and *miR-335* ^*+/Neo*^ mice.

### Expressions of *H19* and *Igf2r*, maternally expressed imprinted genes, are affected in *Mest* ^*+/-*^ *; DMD*-*null* mice

Functions of Mest still remain unknown. Previous studies have demonstrated regulatory networks of imprinted genes [5]. We found that expressions of *H19* and *Igf2r* genes, maternally expressed imprinted genes, were affected significantly in *Mest* ^*+/-*^ *; DMD-null* mice, suggesting that Mest affects these genes during muscle regeneration in *DMD-null* mice. We especially note augmented expression of *H19* in *Mest* ^*+/-*^ *; DMD-null* mice. The *H19* gene is located in a specialized domain in the genome governed by gene imprinting [21]. In this domain, the *H19* gene is co-regulated negatively with the *Igf2* gene by the imprinting center called differentially methylated region (DMR)/ICR localized between these two genes. Interestingly, expressions of both genes were affected just in *DMD-null* mice. The enhanced expression of *H19*, but not that of *Igf2*, in TA muscles of *DMD-null* mice was further augmented in *Mest* ^*+/-*^ *; DMD-null* mice, implying that Mest suppresses the upregulation of *H19* during muscle regeneration in *DMD-null* mice. Mice lac king H19 show overgrowth phenotype while overexpression of H19 results in postnatal growth retardation [6,18]. Taking these previous studies into account, enhanced expression of *H19* in *Mest* ^*+/-*^ *; DMD-null* mice might be associated with their reduced body growth after 4 weeks compared to *DMD-null* mice.

*H19* is a long non-coding RNA that produces no known protein in mice. Venkatraman et al. showed that maternal deletion of the DMR upstream of the *H19* gene results in depletion of H19, which reduces the quiescence of hematopoietic stem cells and activation of Igf2-Igf1r signaling [22]. On the other hand, Kallen et al. showed that H19 antagonizes let-7 miRNAs; either depletion of H19 or overexpression of let-7 causes precocious muscle differentiation in cultured C2C12 myoblasts [23]. Thus, imprinting gene network including *H19* and *Igf2* likely affects maintenance and differentiation of muscle stem cells.

In this study, we could not assign any roles to miR-335 in skeletal muscle development and regeneration *in vivo*. It has been reported that miR-335 suppresses breast cancer metastasis by targeting *SOX4* and *Tenascin C* [24], small cell lung cancer metastasis by targeting *IGF1R* and *RANKL* [25], and induces apoptosis of breast cancer cells by regulating *ERα*, *IGF1R*, *SP1*, and *ID4* [26]. miR-335 is also involved in the induction of p53 tumor suppressor pathway by targeting *Rb-1* [27], and regulates cell proliferation and differentiation of mesenchymal stem cells by targeting *RUNX2* [28]. Thus, roles and functions of miR-335 likely vary in different contexts. Analyses of miR-335-deficient mice we generated would elucidate physiological or pathological significance of miR-335 *in vivo*.

## Materials and Methods

### Ethical Statement

This study was carried out according to the Regulations of Animal Experimentation at Kyoto University. The protocol was approved by the Animal Research Committee of Kyoto University (Permit Number: J-6, J-7). CTX injections were performed under anesthesia using isoflurane inhalation. Mice were sacrificed by cervical dislocation prior to tissue collection, and all efforts were made to minimize suffering.

### Mice

Generation of *Mest* ^*+/-*^ (maternal/paternal) mice was reported previously [2]. *Mest* ^*+/-*^ mice were backcrossed 4–7 times with C57BL/6 mice. CAG-*cre* transgenic [29] mice expressing Cre recombinase under the control of the CAG (CMV enhancer and chicken β-actin promoter) and *DMD-null* [12] mice were kindly provided by Dr. Jun-ichi Miyazaki and Dr. Kazunori Hanaoka, respectively. *DMD-null* mice were crossed with and maintained on a C57BL/6 background. *miR-335* ^*+/Neo*^ mice (Accession No. CDB1107K: http://www.clst.riken.jp/arg/mutant%20mice%20list.html) were produced as follows and were backcrossed 1–4 times with C57BL/6 mice. To construct a miR-335 targeting vector, we inserted a floxed neomycin-resistance cassette (fNeo) into the miR-335 genomic locus using multisite Gateway system (Invitrogen). First, we generated three independent constructs of pENTR-fNeo, pENTR-5’, and pENTR-3’ with the latter two containing miR-335 genomic sequences. For the generation of pENTR-fNeo, fNeo fragment was amplified from pBS-fNeo plasmid by PCR and cloned into the pDONR221 using BP Clonase II enzyme (Invitrogen). For pENTR-5’ sequence of miR-335, the 8.0 kb of 5’ arm was retrieved from BAC DNA RP24-211G11 (CHORI) by using amplified DNA derived from pDONR P4-P1R. For ENTR-3’ sequence of miR-335, the 2.5 kb of 3’ arm was amplified from BAC DNA RP24-211G11 by PCR and cloned into the pDONR P2R-P3 using BP Clonase II enzyme. Second, the three plasmids were cloned into pDEST R4-R3 plasmid using LR Clonase II Plus enzyme (Invitrogen). The miR-335 targeting vector was linearized and introduced into TT2 ES cells by electroporation [30]. Briefly, ES cells were trypsinized and suspended at a concentration of 1x10^7^/ml in 0.4 ml HBS. Sixty nM of linearized DNA in 0.1 ml HBS was added to the ES cell suspension and mixed, followed by a single pulse of electroporation at room temperature using Bio-Rad Gene Pulser II (0.8 kV, 3.0 uF). Screening of ES cells with homologous recombination and production of chimera mice were performed as described (http://www.clst.riken.jp/arg/Methods.html). Briefly, ES cells with homologous recombination at the target site were screened by PCR. Homologous recombination was confirmed by Southern blotting with 5’, 3’, and neomycin probe, prepared using DIG DNA labeling mix (Roche) (Fig 2B). The primers used to generate the probes 5’, 3’, and neomycin are shown in S1 Table. The 5’ probe that was amplified from BAC DNA RP24-211G11 by PCR detected a 16.2 kb and a 12.7 kb band for WT and the targeted allele, respectively, when genomic DNA was digested with BamHI. The 3’ probe that was amplified from genomic DNA by PCR detected a 13.2 kb and a 3.4 kb band for WT and the targeted allele, respectively, when genomic DNA was digested with HindIII. The neomycin probe that was amplified from pBS-fNeo plasmid detected a 5.0 kb band for the targeted allele of genomic DNA digested with BamHI. The established mice, *miR-335* ^*+/Neo*^, were crossed with CAG-*cre* transgenic mice to delete neomycin resistance cassette (*miR-335* ^*+/-*^, Fig 2A). Sequences of the primers are listed in S1 Table. For P0 mice, gender was determined by the presence or absence of *Sry* gene, assessed by PCR of genomic DNA in tails (Forward: 5’ TTGTCTAGAGAGCATGGAGGGCCATGTCAA3’ Reverse: 5’CCACTCCTCTGTGACACT TTAGCCCTCCGA3’, http://mgc.wustl.edu/Protocols/PCRGenotypingPrimerPairs/tabid/154/Default.aspx).

### Southern Blotting

Southern blotting was performed as follows. Briefly, genomic DNA that was isolated from ES cells and digested with BamHI or HindIII was resolved on 0.7% agarose gels, transferred to Hybond-N+ membrane (GE Healthcare Life Sciences), and was exposed under the UV light using a UV crosslinker (FUNA-UV-LINKER FS-800, Funakoshi). Next, the membrane was hybridized with 5’, 3’, and neomycin probes labeled with DIG-dUTP at 40°C overnight, incubated with stringency solution and hybridized with Anti-Digoxigenin-AP, Fab fragments (Roche). Signals were detected using CDP-Star (Roche) in automatic processor (Fujifilm).

### PCR

Genomic DNA in tails was isolated using 100 μg/ml Proteinase K in lysis buffer (150 mM NaCl, 10 mM Tris-HCl pH 8.0, 10 mM EDTA, and 0.5% SDS) at 55°C overnight. PCR was performed using KOD FX (TOYOBO) according to the manufacture’s instructions. Briefly, genomic DNA including reaction mixture preheated to the denaturation temperature (94°C) for 2 minutes was amplified in denaturation (98°C) for 15 seconds, annealing (58–60°C) for 30 seconds, and extention (68°C) for 60 seconds/kb of expected product in thermocycler (Biometra). The number of denaturation-annealing-extention cycle was 35.

### qRT-PCR analyses

Total RNA was isolated from skeletal muscles that were treated in 0.2% Collagenase Type 2 (Worthington) using miRNeasy Micro Kit (QIAGEN) according to the manufacture’s instructions. For analyses of mRNA expression, cDNA synthesis with oligo dT primers (Invitrogen) was performed using SuperScript III Reverse Transcriptase (Invitrogen). For analyses of miR-335 expression, reverse transcription was performed using TaqMan MicroRNA Reverse Transcription Kit (Applied Biosystems) according to the manufacture’s instructions. qRT-PCR was performed using Power SYBR Green PCR Master Mix for mRNAs or TaqMan Universal PCR Master Mix (Applied Biosystems) for miRNAs on StepOnePlus (Applied Biosystems). Sequences of the primers are listed in S2 Table [5,31–33], snoRNA-202 (Assay ID: 001232, Applied Biosystems) and miR-335 (Assay ID: 000546, Applied Biosystems).

### Histological analysis

To cause the injury in TA muscles of 8–15 weeks old male mice (n = 36), 100 μl of 10 μM CTX (SIGMA) was injected into TA muscles under isoflurane anesthesia (Mylan). TA muscles were excised and frozen in liquid nitrogen-cooled isopentane (Nacalai Tesque). Transverse sections (10 μm) were cut using cryostats (CM3050S, Leica Microsystems) and collected onto MAS coated glass slides (Matsunami). H&E staining was carried out on these sections. Photomicrographs were obtained with a microscope (BX50), a digital camera (DP72) and DP2 BSW software (all from Olympus). Cross Section Areas (CSA) (Fig 4B) was evaluated on pictures showing H&E stained skeletal muscle using DP2 BSW software. CSA (Fig 4D) was evaluated on pictures showing Laminin-stained (not shown) skeletal muscle using ImageJ. The number of myofibers was counted on pictures showing Laminin-stained (not shown) skeletal muscles using Keyence analysis application.

### Immunohistochemistry

Sections of TA muscles were fixed in 4% PFA, permeabilized with cold methanol, blocked with 50 mM NH_4_Cl, reacted with 0.01 M citric acid (pH 6.0) at 80°C for 10 minutes for antigen retrieval, and blocked in M.O.M. kit (Vectorlabs). Primary antibodies against Pax7 (Mouse, 1/200, Developmental Studies Hybridoma Bank) and Laminin α2 (Rat, 1/400, ALEXIS) were used. Secondary antibodies conjugated with Alexa Fluor 488 or 594 (1/500, Molecular Probe) were used. DAPI was used to stain nucleic acid. Fluorescence was obtained with fluorescence microscopes (AF6000: Leica Microsystems and BIOREVO: Keyence).

### Statistical analysis

Each error bars indicate standard error of the mean (s.e.m.). Statistical analyses were carried out using Student’s t test between two groups and Dunnett’s test among more than two groups. A value of *P* < 0.05 was considered statistically significant unless otherwise specified.

## Supporting Information

S1 Fig The ratio of body weight. The ratio in body weights of male littermate mutant mice from 1 to 12 (11–13) weeks old.
(TIF)

S2 Fig *Pax7* and *MyoD* expression are not affected in CTX injured TA muscles of *Mest* ^*+/-*^ mice. qRT-PCRs for *Pax7* and *MyoD* mRNA were performed in TA muscles of 12–15 weeks old WT (n = 5) and *Mest* ^*+/-*^ mice (n = 3) 6 days after CTX. Error bars indicate the s.e.m.
(TIF)

S1 Table Primer list for generation of miR-335 deficient mice. (DOCX)

S2 Table Primer list for qRT-PCR. (DOCX)
