# Supplementary figures and images for: Mest but Not MiR-335 Affects Skeletal Muscle Growth and Regeneration
Source: PLoS One. 2015 Jun 22;10(6):e0130436. doi: 10.1371/journal.pone.0130436 (PMC4476715; doi:10.1371/journal.pone.0130436)

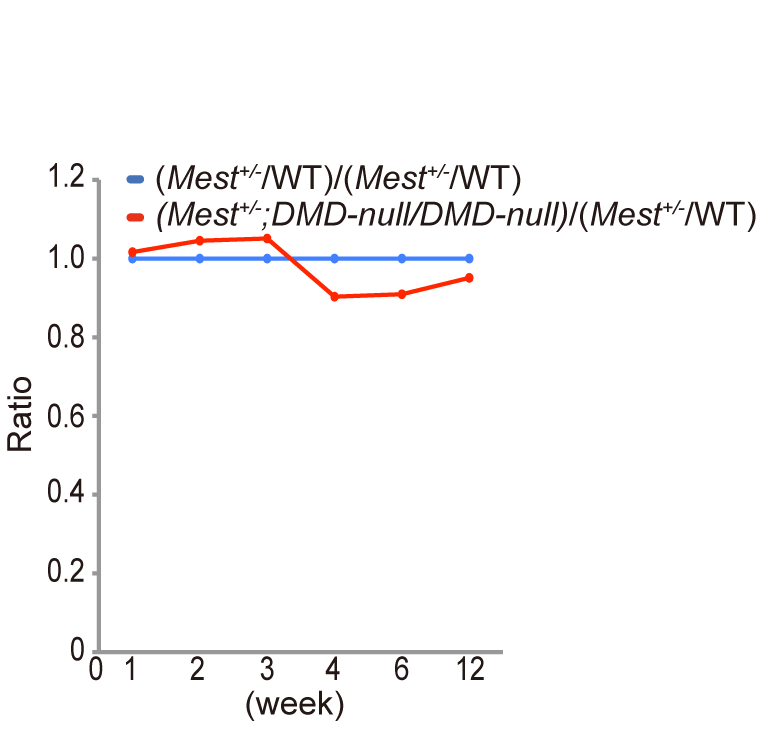

Supplement: S1 Fig — The ratio in body weights of male littermate mutant mice from 1 to 12 (11–13) weeks old. (TIF) [file pone.0130436.s001.tif]

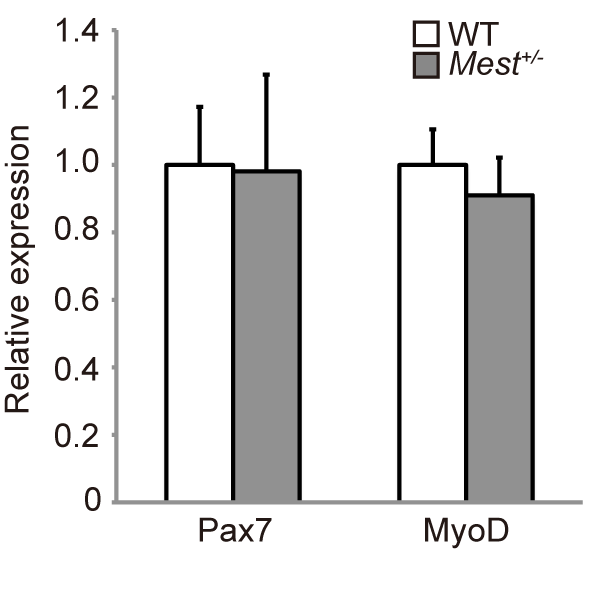

Supplement: S2 Fig — qRT-PCRs for Pax7 and MyoD mRNA were performed in TA muscles of 12–15 weeks old WT (n = 5) and Mest +/- mice (n = 3) 6 days after CTX. Error bars indicate the s.e.m. (TIF) [file pone.0130436.s002.tif]

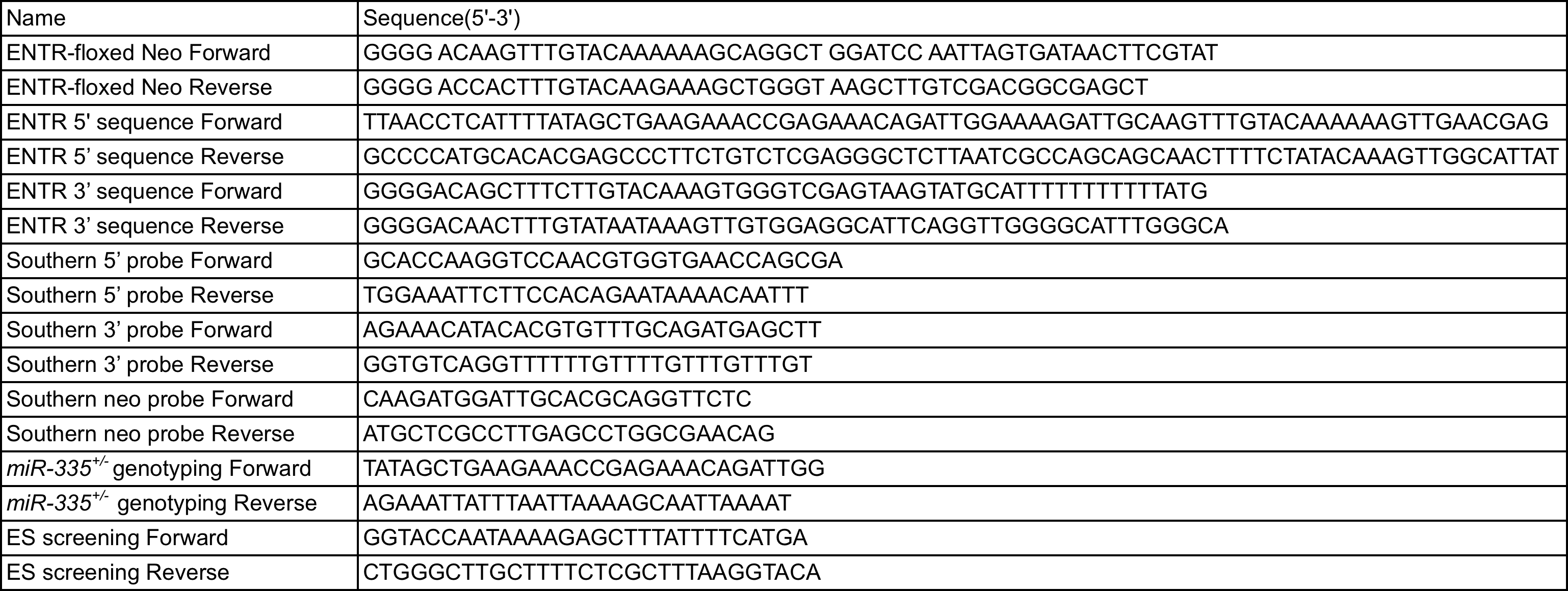

Supplement: S1 Table — (DOCX) [file pone.0130436.s003.docx]
