# Supplementary material for: Mest but Not MiR-335 Affects Skeletal Muscle Growth and Regeneration
Source: PLoS One. 2015 Jun 22;10(6):e0130436. doi: 10.1371/journal.pone.0130436 (PMC4476715; doi:10.1371/journal.pone.0130436)
Supplement: S2 Table — (DOCX) [file pone.0130436.s004.docx]

| Gene |  | Sequence (5'-3') |
| --- | --- | --- |
| Gapdh | Forward | GGCAAAGTGGAGATTGTTGC |
|  | Reverse | AATTTGCCGTGAGTGGAGTC |
| Mest | Forward | GAAATTCAGAAGACGCTGGGTGGG |
|  | Reverse | CTCCAAAAACTCTGGATACG |
| Igf2 | Forward | GCTTGTTGACACGCTTCAGTTTG |
|  | Reverse | GTTGGCACGGCTTGAAGGC |
| Peg3 | Forward | TTGGACTGGACAGAGATGATGACA |
|  | Reverse | ATTCTGGTATGACTCGGCATCCT |
| Zac1 | Forward | TTTTCTTTGCCTAGCTTAACCTACTACTT |
|  | Reverse | CACAATCCTCTTGGGATACAAACTAA |
| Dlk1 | Forward | CTGGAGAAAGGCCAGTACGA |
|  | Reverse | AGGGAGAACCATTGATCACG |
| H19 | Forward | CTTGTCGTAGAAGCCGTCTGTTC |
|  | Reverse | GTAGCACCATTTCTTTCATCTTGAGG |
| Grb10 | Forward | AGGATCATCAAGCAACAAGGTCTC |
|  | Reverse | ATTACTCTGGCTGTCACGAAGGA |
| Igf2r | Forward | GCACAGAATCCAGACTAGCATTACA |
|  | Reverse | CTCCTTATCAGCTTTAAATATGTCTTTCTT |
| Pax7 | Forward | AGGCCTTCGAGAGGACCCAC |
|  | Reverse | CTGAACCAGACCTGGACGCG |
| MyoD | Forward | AGCACTACAGTGGCGACTCA |
|  | Reverse | GGCCGCTGTAATCCATCAT |
